# Application of 3D Printing in the Design of Functional Gluten-Free Dough

**DOI:** 10.3390/foods11111555

**Published:** 2022-05-25

**Authors:** Adrián Matas, Marta Igual, Purificación García-Segovia, Javier Martínez-Monzó

**Affiliations:** Food Technology Department, Universitat Politècnica de València, Camino de Vera s/n, 46022 Valencia, Spain; admagi@doctor.upv.es (A.M.); marigra@upvnet.upv.es (M.I.); xmartine@tal.upv.es (J.M.-M.)

**Keywords:** 3D food printing, gluten-free, dough, rosehip, functional and technological properties

## Abstract

The design of functional foods through 3D printing is proposed here as one of the most appropriate technologies to provide closer food personalization for the population. However, it is essential to study the properties of the biomaterials intended to be printed. This work will evaluate the incorporation of rosehip as a functional ingredient in a gluten-free dough. Three types of dough (control, rosehip, and encapsulated rosehip) were printed in a rectangular figure of dimensions 7 cm long, 2 cm wide, and 1, 2, and 3 cm high. Changes in printed figures before and after baking were evaluated by image analysis. Physicochemical properties, total phenols (TP), antioxidant capacity (AC), and total carotenoids (TC) were determined both in the pre-printed doughs and in the printed and baked samples. The bread enriched with rosehips presented more orange colors in dough and crumbs. They were also more acidic than control, probably due to the ascorbic acid content of rosehip. The addition of rosehip generally makes the product more resistant to breakage, which could be due to the fiber content of the rosehip. It was observed that the incorporation of rosehip notably improved the functional properties of the bread.

## 1. Introduction

Food products are critical factors for human health and well-being and are constantly innovated. The relationship between food, nutrition, and health is multifaceted and affected by significant factors [1]. Globally, diet-related chronic diseases have increased despite public health population-based strategies and recommendations. In the last few decades, a new concept emerged as an effective alternative strategy for prevention which has been referred to as “personalized nutrition”. Personalized nutrition is postulated as individual guidelines based on specific needs to promote dietary patterns and obtain health benefits [1]. Personalization stands out among the current food design trends based on the close relationship between nutrition and food.

3D printing is an emerging and valuable technique used to personalize food and valorized food byproducts [2,3]. However, the major challenge in the formulation of printable food-inks is flowability through the nozzle, printing stability, and structure retention during the printing [4]. Many printable materials, such as cookies [3,5], snacks [6], gels [7,8,9], chocolate [10], peanut butter, and cream cheese [9], have been studied in the last ten years. 3D printing has been used to develop functional foods and/or manufacture new shapes, textures, flavors, and colors [11,12].

Since the 1980s, the development of foods to improve the population’s quality of life and reduce the risk of illness has become a trend in food companies. ‘Functional foods’ is currently a well-known concept used in scientific or social media to refer to foods that may provide added functional benefits to consumers [13]. Gluten-free products are also included in functional foods, as eliminating gluten from their composition decreases the risk of a disease state in the celiac population. Although these products support the daily life of the gluten-intolerant group, it has been observed that strict adherence to a gluten-free diet leads to certain nutritional deficiencies [14]. The primary deficiencies are in micronutrients such as B vitamins, especially folic acid, vitamin D, calcium, and iron, and macronutrients such as fiber. Therefore, this population is at an increased risk of cardiovascular and metabolic diseases, diabetes, overweight, and obesity [14]. According to these needs, gluten-free foods must be enriched with other ingredients that supplement these nutrients.

Nowadays, there is a growing interest in wild plants as they have a high content of bioactive compounds that positively affect health. Rosehip (Rosa canina) have been used for medicinal remedies for years. A high concentration of vitamin C and flavonoids, tannins, and vitamins A, B1, B2, B3, and K are found in this fruit [15]. Recent studies have shown that the components of rosehip are dominated by vitamin C, proanthocyanins, galactolipids, and folate. In addition, flavonoids, pectin, vitamin A and other B-complex vitamins, vitamin E and minerals such as Ca, Mg, K, S, Si, Fe, Se, and Mn are found [16].

It should be noted that rosehip has a moderate content of carotenoids, 224 mg/kg of boneless fruit, mainly consisting of lycopene and β-carotene [17]. Carotenoids are bioactive compounds with beneficial properties for health, such as their antioxidant function [18,19]. Rosehips have already been used to enhance snacks using the filtrate from the pulp [20] and the co-product formed by the fibrous walls [21]. Its antioxidant power has also been compared with sodium nitrite and sodium ascorbate in frankfurters [22].

However, to incorporate functional ingredients into foods, they often undergo a treatment to extract the desired compounds to obtain a final liquid or powdered ingredient. Bioactive compounds, as described above, can be degraded under certain environmental conditions, such as high pressures and temperatures, during the extraction process. Microencapsulation is recognized as a tool to improve the delivery of bioactive compounds into foods [23], particularly described in rosehip. Biopolymers such as cyclodextrins [24], maltodextrin [21] or pea protein [25] are often incorporated as encapsulation agents of bioactive ingredients to protect them.

Personalization of food is one of the new food trends where 3D printing can be attractive by its capability of building complex structures and textures. Considering this, the main goals of this work were: (i) to study the physical-chemical and rheological properties of gluten-free bread dough to evaluate 3D printability; (ii) to study printing stability and structure retention during the printing and after baking bread; and (iii) to obtain a personalized gluten-free bread enriching with rosehip as a source of bioactive compounds.

## 2. Materials and Methods

### 2.1. Materials

Gluten-free flour preparation was supplied from Sinblat (Sinblat Alimentación Saludable S.L., Foios, Spain). Salt, water, baking powder, and oil were purchased from a local supermarket. Rosehip (*Rosa canina*) was collected in September–October 2020 in Aldehuela (Teruel, Spain).

### 2.2. Rosehip Powder Preparation

Rosehip fruits were washed and homogenized with a Thermomix (TM 21, Vorwerk, Valencia, Spain) for 1 min at 5200 rpm. Then, 1000 g of distilled water were added and homogenized for 5 min at 5200 rpm. The mixture was filtered with a sieve (1 mm diameter, Cisar 029077, series). Two formulations were prepared: unmodified rosehip (R) from the mixture and encapsulated rosehip (RM) with maltodextrin. In RM samples, 10 g maltodextrin was added to 90 g of the filtered mixture. Samples were frozen at −42 °C in an upright freezer (CVF450/45, Ing. Climas, Barcelona, Spain) for 24 h in aluminum trays (15 cm diameter and 5 cm high) and after lyophilized in a Lioalfa-6 Freeze-Dryer (Telstar, Barcelona, Spain) at 2600 Pa and −56.6 °C for 48 h. The product obtained was crushed in a mill (Minimoka, Taurus, Lleida, Spain) to obtain the powder to incorporate into the dough.

### 2.3. Dough Preparation

Three different doughs were formulated, control (CD), rosehip (RD), and rosehip en-capsulated with maltodextrin (RMD). All doughs were composed of 56% water, 40% gluten-free flour, 2.4% baking powder, 1.2% oil, and 0.4% salt. In those with rosehip powder (R and RM), 7% were added subtracted from the gluten-free flour amount. These ingredients were mixed (Kenwood chef classic, KM400/99 plus, Kenwood Corpo-ration, Tokyo, Japan) at minimum speed for 45 s followed by 5 min at speed 2. Each dough was transferred to a syringe for 3D printing.

### 2.4. 3D Printing Assay

A 3D food printer (BCN 3D+, BCN3D Technologies, Barcelona, Spain) equipped with a pasta extruder nozzle designed for food materials (BCN3D Technologies, Barcelona, Spain) was used for printing. The 3D printing system consisted of an extrusion system (syringe plus pump) and an X-Y-Z positioning system using stepper motors. Printing was done at room temperature, with a 3 mL/min extrusion rate and nozzle diameter of 1.63 mm. A stainless-steel plate was used as support in the following baking process. Three different figures were designed, all of them with a rectangular base of 7 cm long, 3 cm wide but variable height, 1, 2, and 3 cm, using Autodesk 123D design program (Auto-desk Inc., San Rafael). These figures were introduced in the Slic3r program (Alessandro Ranellucci) to configure the printing parameters: nozzle speed 20 mm/s, layer height 1.7 mm, and 60% rectilinear infill except for the first layer (100% infill).

### 2.5. Baking and Postprocessing

Samples were baked at 190 °C (Convotherm mini, Welbilt Iberia, Barcelona, Spain), and the time of baking was adapted to the height of the sample, 16, 22, and 26 min for 1, 2, and 3 cm, respectively. After, samples were cooled at 25 °C prior to analytical determinations.

### 2.6. Determinations

#### 2.6.1. Rheological Properties of Dough

The rheological properties of doughs (control, with rosehips and rosehips and maltodextrin) were characterized on a Kinexus Pro + rotational rheometer (Malvern Instruments, Worcestershire, UK). A 25 mm plate-plate oscillatory test was carried out with 2 mm gap between plates, 1 Pa stress, a frequency range of 0.1 to 10 Hz [26,27] and a heat-controlled sample stage (Peltier Cylinder Cartridge, Malvern Instruments, Worcestershire, UK) at 25 °C. Preliminary tests indicated that the strain was well within the linear visco-elastic region.

Rheological properties can be closely associated with the extrusion printability of doughs. The elastic or storage modulus (G′) characterizes the solid-like component, and the viscous or loss modulus (G″) describes the liquid-like behavior [28,29]. The relation between loss modulus and storage modulus is represented by the loss factor (tan δ = G″/G′) where a high value of tan δ is indicative of a predominant viscous behavior in materials, and low values (tan δ < 1) indicate a predominant elastic behavior. The complex viscosity η* (Pa s), defined as the material’s resistance to flow in the structured state, was also calculated [30]. The viscoelastic behavior of the dough can indicate if it will have a stable printed shape and if the dough is suitable for extrusion, respectively [31]

#### 2.6.2. Image Analysis

**Figure 1:**
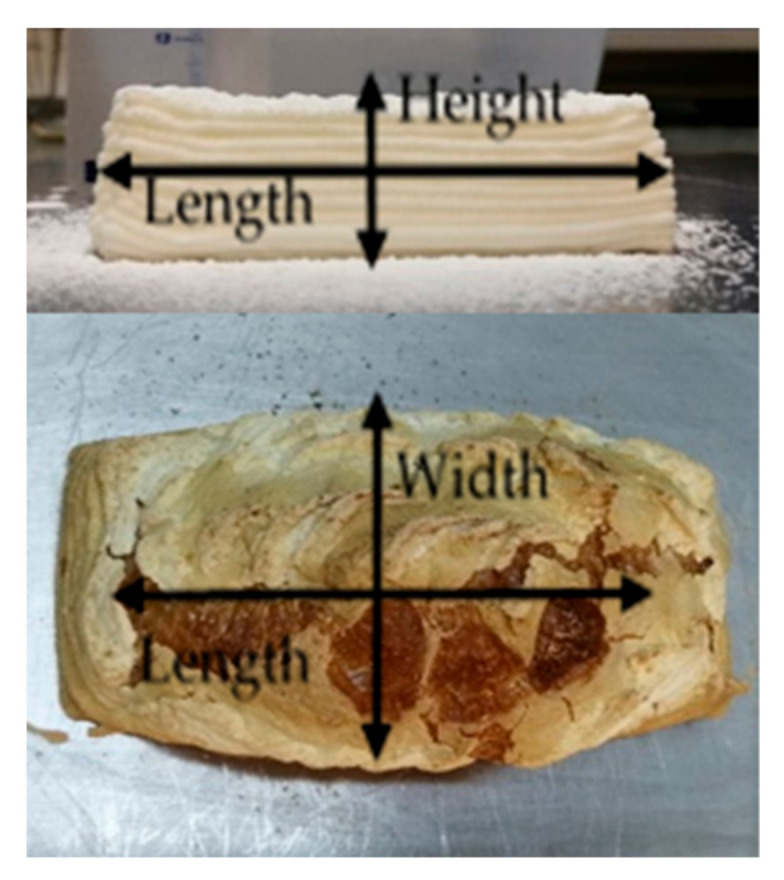
Sample data acquisition in image analysis.

Pictures of each bread’s top and lateral view were taken before and after baking. These images were processed in ImageJ program (ImageJ, NIH, Washington, DC, USA) to determine the shape evolution before and after baking. For top view, length and width were measured; for lateral view, width and height were measured (Figure 1). As a metric of variation of each dimension, differences between printed and baked figures were calculated.

#### 2.6.3. Physico-Chemical Parameters

Water activity was determined using a hygrometer (AquaLab PRE, Decagon De-vices, Inc., Pullman, WA, USA). Baked samples and doughs were quantified with a pH meter (MM41 MultiMeter, Crison Instruments, S.A., Barcelona, Spain). The moisture (g/100 g) of samples was determined by vacuum drying in a vacuum oven (Vaciotem, J.P. Selecta, Spain) at 70 ± 1 °C and below 100 mmHg pressure until constant weight [32].

#### 2.6.4. Colour Measurement

CIE L * a * b * coordinates (L *: luminosity, a *: green/red abscissa axis, b *: yellow/blue ordinate axis) were measured using a Konica Minolta CM-700d colourimeter (Konica Minolta Sensing, Inc., Tokyo, Japan) with a SAV aperture size accessory of 6 mm. Measurements were made with illuminant D65 and a visual angle of 10° for bread crust and crumbs. Colourimetric parameters, tone (h), chroma (C), and color difference (ΔE) were calculated.

#### 2.6.5. Textural Characterization

TPA and puncture tests were carried out using a TA.XT.plus texturometer (Stable Micro Systems, Godalming, Surrey, UK) and Texture Exponent 32 program (Stable Micro Systems, Godalming, Surrey, UK) was used for data analysis. The TPA of the crumb was executed with a cylindrical aluminium probe (4 cm in diameter) using a 50 kg load cell. The crosshead speed was 0.5 mm/s, with a rest period of 5 s between cycles and the deformation was 40% of the original length. Six textural parameters were determined from each curve: hardness (peak force of the first compression cycle in N), adhesiveness (negative area under the baseline between the compression cycles in N s), cohesiveness (ratio of positive force area during the second compression compared to that during the first compression, dimensionless), springiness (ratio of the time duration of force input during the second compression to that obtained during the first compression, dimensionless), chewiness (hardness multiplied by cohesiveness multiplied by springiness in N), and resilience the ratio of the area under the curve of the second half of the first cycle to the first half [33].

A puncture test was carried out using a 2 mm probe with a crosshead speed of 0.6 mm/s. The force-time curve was registered, and maximum puncture force (N) was obtained.

#### 2.6.6. Centesimal Composition

Proteins, fat, available carbohydrates, moisture, ashes, and fiber were quantified to obtain the centesimal composition of the samples.

The nitrogen content was determined by Dumas’s method according to AOAC method 990.03 [32]. The Leco CN628 elemental Analyzer (Leco Corporation, St. Joseph, MI, USA) was used for this essay. Crude protein (CP) was calculated as the nitrogen con-tent multiplied by nitrogen factor (5.62) used for corn [34].

ANKOM methodology (ANKOM Technology, Macedon, NY, USA) was carried out for fat determination. Heat-sealed cellulose sachets were filled with 0.5 g of the freeze-dried samples and introduced into the ANKOM equipment (ANKOM Technology, Macedon, NY, USA). After the essay, the cellulose sachets were stored in an oven for 24 h to measure the weight difference later.

The ash content was measured following the method 930.05 of the AOAC [32]. Approximately 0.5 g of the freeze-dried bread were introduced into a muffle (Select-Horn, Selecta, Barcelona, Spain) and ramped up to 550 °C, where it was maintained for 24 h. Once the muffle furnace had cooled, the tempered crucibles were weighed to calculate the ashes by difference.

The free carbohydrates determination was made by anthrone method [35,36]. A calibration line was developed with 0, 10, 25, 50, 75 and 100 mg/L of D-glucose standard, an-throne reagent 1 g/L was prepared with 96% sulfuric acid. One gram of each freeze-dried sample was taken and mixed with 10 mL of deionized water and 15 mL of 52% perchloric acid and left for 12 h. Then, it was made up to 100 mL, filtered and made up to 250 mL. A 10 mL aliquot was taken, made up to 100 mL, and measured in the UV-3100PC spectrophotometer (VWR, Leuven, Belgium) at 620 nm.

The moisture determination was carried out as described in the “physicochemical analysis” section. Finally, fibre was calculated as a difference from the rest of the components.

#### 2.6.7. Bioactive Compounds

Bioactive compounds were determined from dough and bread samples after freeze-dried. The assays consisted in the determination of total phenols (TP), antioxidant capacity (AC) and total carotenoids (TC).

Total phenols’ methodology explained in Garcia-Segovia et al. [37] was followed with some modifications. One gram of freeze-dried sample was homogenized at 10,000 rpm for 2 min using an ultra-turrax (Ultra-Turrax T25, Janke & Kunkel IKA-Labortechnik, Staufen, Germany) with 5 mL of methanol and centrifuged (Eppendorf Centrifuge 5804 R, Hamburg, Germany) at 4 °C and 10,000 rpm for 10 min. Then, 250 μL of the supernatant was pipetted to a volumetric flask and added 1.25 mL of Folin-Ciocalteu reagent (Sigma-Aldrich, Steinheim, Germany) and stored in a place isolated from light for 8 min. Later, 3.75 mL of sodium carbonate were added, and the mix was stored again for 2 h. Finally, samples were placed in plastic cuvettes and analysed on the UV-3100PC spectrophotometer (VWR, Leuven, Belgium) at a wavelength of 765 nm. The results were expressed as mg of gallic acid (Sigma-Aldrich, Steinheim, Germany) equivalent per 100 g of sample.

The determination of antioxidant capacity (AC) was carried out as Igual et al. [38] described. This method assesses AC by neutralizing free radicals in samples with DPPH (2, 2-diphenyl-1-picrylhydrazyl). A volume of 0.1 mL was taken from the previous supernatant and added 3.9 mL of DPPH (0.030 g/L, Sigma-Aldrich, St. Louis, MO, USA) in methanol in plastic cuvettes. The samples were prepared and, after 5 min of reaction, were measured with a spectrophotometer UV-3100PC (VWR, Leuven, Belgium) at 515 nm. The percentage of DPPH was calculated using the following equation (Equation (1)):

(1) $$\%DPPH=\frac{A_{\mathrm{control}}-A_{\mathrm{sample}}}{A_{\mathrm{control}}}\times 100.$$

where, A_control_ is the absorbance at initial time and A_sample_ is the absorbance after 5 min.

The results were expressed as millimoles of Trolox equivalents (TE) per 100 g (mg TEq/100 g) using a Trolox calibration curve in the range of 10–500 mg/L (Sigma-Aldrich, Steinheim, Germany).

Finally, the quantification of total carotenoids was carried out by spectrophotometry as indicated in AOAC [32]. Following the extraction methodology of Olives-Barba et al. [39]. Five grams of the samples were taken, a mixture of hexane/acetone/ethanol (50:25:25 *v*/*v*/*v*) was incorporated and shaken in darkness for 30 min. Then, 15 mL of deionized water were added, and 4 mL of the upper phase (hexane phase) was taken and placed in a glass cuvette. The sample was measured on the UV-3100PC spectrophotometer (VWR, Leuven, Belgium) at 446 nm. The results were expressed as mg of β-carotene/100 g.

### 2.7. Statistical Analysis

Analysis of variance (ANOVA) was performed for a 95% confidence interval (*p* < 0.05) to evaluate the differences between the different bread. In addition, a multivariate analysis was performed to see the possible interaction between height and dough type. All of these analyses were carried out with the Statgraphics Centurion 18 program, version 18.1.13 (Statgraphics Technologies, Inc., The Plains, VA, USA).

## 3. Results and Discussion

### 3.1. Rheological Characterisation of Dough

Table 1 shows the results of these parameters for the three doughs studied. Data values at 1 Hz were used to compare the results. As 3D printing allows personalization, rosehip (R) and encapsulated rosehip (RM), as a source of bioactive compounds, were added to the dough to evaluate changes in rheological properties [40,41]. There were no significant differences in G′, G″ and η* between control (C) and encapsulated rosehip (RM) dough.

**Figure 2:**
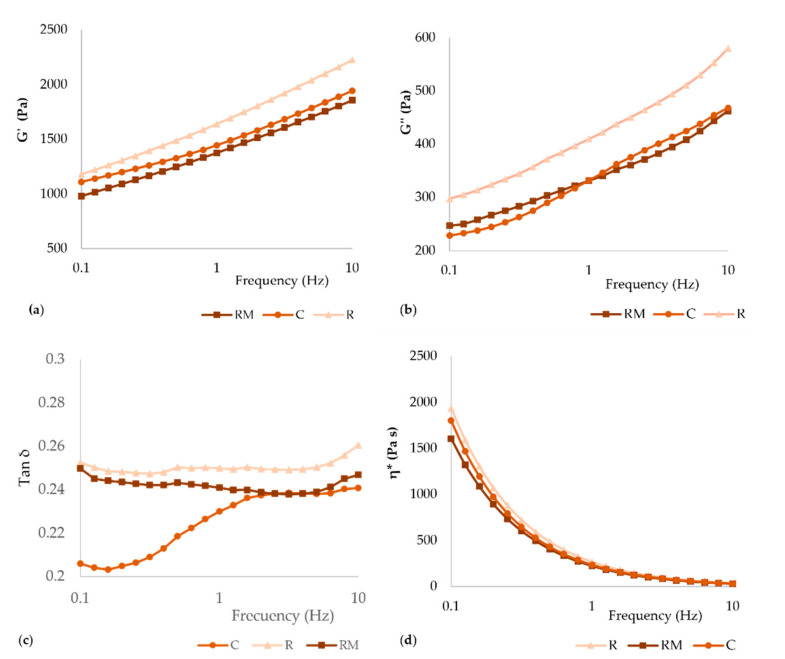
Rheological properties of gluten-free dough: (**a**) storage modulus G′, (**b**) loss modulus G″, (**c**) loss factor tan δ, and (**d**) complex viscosity versus frequency (Hz). (C, control; R, rosehip; RM, encapsulated rosehip).

Figure 2 represents elastic and viscous modulus, tan δ, and complex viscosity of doughs versus frequency profile in Hz. For all samples, elastic modulus (G′) was greater than viscous modulus (G″), which suggests a solid elastic-like behavior of the gluten-free doughs (Figure 2a,b). The elastic (G′) and viscous (G″) moduli were higher in R dough. This increased resistance may be related to the rosehip’s fiber [39]. The incorporation of encapsulated rosehip did not modify the resistance concerning the control sample. This effect could be due to the lower proportion of fiber in this sample.

The tan δ < 1 indicated a predominant elastic behavior in all doughs (Figure 2c). Similar results were reported previously for wheat doughs [42], rice flour [43] and gluten-free mixes [28]. This behavior could indicate that doughs had a stable shape after print, as discussed next. The dough must have sufficient elasticity to be printed [40]. These results align with other studies and applications using alternative flour mixes to limit the dough elasticity that distorts the extruded filament on printed 3D structures [40,44,45]. A decrease in complex viscosity over frequency was observed for all gluten-free dough samples. Similar findings were reported in a previous studies with gluten-free formulation [46,47,48]. The addition of encapsulated rosehip (RM) showed no significant difference compared to the control. The addition of rosehip (R) exhibited only significant differences at low frequency (frequency < 1).

### 3.2. Image Analysis

**Figure 3:**
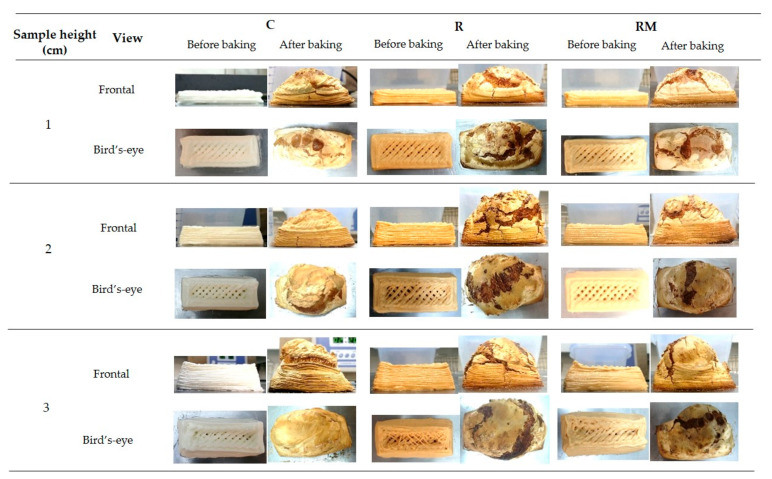
3D printed samples front and bird’s-eye view before and after baking process. (C, control; R, rosehip; RM, encapsulated rosehip).

An image assay was performed to know the precision of the 3D printer and the dough’s aptitude to maintain the desired shape. Height, width, and length were measured before and after baked, as shown in Figure 1. Figure 3 visually compares dough and baked samples with different heights and compositions. It could be seen that the printed samples showed the different lines forming each layer. These lines were evident, giving the product’s structure a stable appearance. The dimensions of the samples changed after baking, mainly the height increased. After baking, deposition lines were deformed and disappeared. It was evident in upper area infill lines (Figure 3 bird’s-eye view).

**Figure 4:**
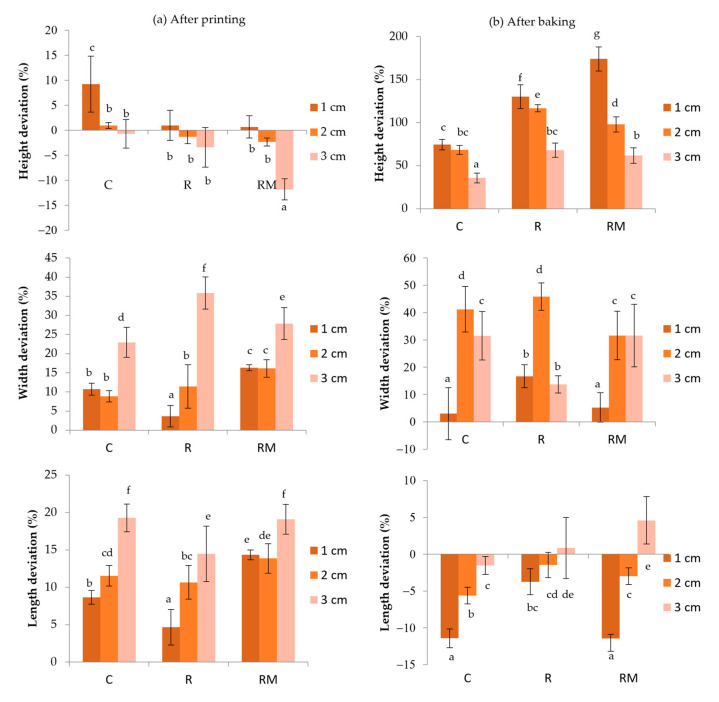
(**a**) Deviations of the proportions after printing samples; (**b**) Deviations of the proportions after baking samples (C, control; R, rosehip; RM, rosehip encapsulated; 1, 2, 3: height cm). Letters (a–g) indicate homogeneous group according to ANOVA (*p* < 0.05).

Figure 4a shows the deviation (%) of the shape parameters (7 cm length, 3 cm width and 1, 2 or 3 cm height) in printed and baked samples. The samples that showed the highest deviation were 3 cm in height. The dimension with more significant deformation was the width; 3 cm R samples had higher deformations.

After printing, samples were baked, and significant changes were observed (Figure 3). After baking, image analysis is critical in bakery products to study the expansion of samples. Different composition samples can influence the expansion parameters.

Figure 4b shows the shape parameters’ deviation after baking printed samples. The samples with the most significant deviation in height were the 1 cm height samples with rosehip (R and RM). Those with the slightest deviations in height were the 3 cm height samples, the lowest in control. In terms of width, the most significant differences were found in the 2 cm ones, with the rosehip and control being the largest. On the other hand, the one with the slightest deviation was the 1 cm control. The ones that deviated the most were the 3 cm control and the encapsulated rosehip. The 1 cm with rosehip showed fewer differences, which could be due to the free rosehip in maintaining the initial printed shape. Finally, the 1 cm control and encapsulated rosehip samples show more significant changes in the length dimension. In these samples, a negative deformation was observed due to an increase in height that produced a shrinkage in shape.

The storage modulus (G′) and damping factor (G″/G′ or tan δ) extracted from an amplitude sweep in oscillatory shear measurements of a food ink are used to characterize rigidity [49]. According to Nijdam et al. [49], the storage modulus (G′) appears in a dimensionless number (ρgH/G′), representing the ratio of deformation force due to food-structure weight to the force countering deformation due to food-ink rigidity. This dimensionless number and the damping factor form the axes of a graph on which a window of dimensional stability is defined, assuming a structural-deformation limit of 5%. This graph shows that food inks with higher damping factors require higher storage moduli to form dimensionally stable 3D structures. Given food-ink rheology, the window allows food inks with appropriate rigidity to be selected or, given food-ink rheology, suitable structural heights to be estimated to form dimensionally stable 3D structures. The graph suggests that, for a given damping factor, the extent of deformation increases as the weight of a structure increases, that is, density (ρ) or height (H) increases. However, as the storage modulus G′ or the elastic portion of rigidity increases for a given damping factor, the extent of deformation decreases. For a given weight (ρgH) and storage modulus G′, the extent of deformation increases as the damping factor G″/G′ increases, which implies that the food ink becomes less solid-like and behaves more like a flowing viscous liquid. As shown in Table 1, G′ values of dough are very similar (between 1408 and 1639 Pa), as well as damping factor (between 0.2300 and 0.2499). With these values and according to Nijdam et al. [49], samples with 1 or 2 cm height can be included in a 5% and 20% deformation tolerances (dimensionless number between 0.7 and 0.14), but 3 cm height samples (dimensionless number 0.234) will be included in a category with more than 20% of deformation. This trend can be observed in Figure 4a were samples with 3 cm height report higher deviation in proportions after printing.

### 3.3. Physicochemical Parameters

In many cases, the study of physicochemical properties allows, in many cases, to know how the new ingredients’ incorporation alters the product’s appearance and/or behavior. The addition of rosehip in dough reduced significantly (*p* < 0.05) the pH values compared to the control dough (6.913 ± 0.006) being, the mean pH values of dough with rosehip was 5.717 ± 0.006 and for dough with encapsulated rosehip 6.387 ± 0.006. The decrease in pH was due to incorporating acids (mostly ascorbic acid [16]) from the rosehip. After baking, the samples followed the same trend but with lower values than doughs. The sample baked with rosehip (4.98 ± 0.03) and the sample with encapsulated rosehip (5.24 ± 0.12) had lower pH than the control (5.847 ± 0.006).

Table 2 shows the water activity (aw) and moisture of the doughs and baked samples. There was no apparent effect in water activity after rosehip addition in the samples. The water content of the samples was higher in those with rosehip and encapsulated rosehip. This increase in water content could be due to the water retention capacity of the fruit fibers [50]. No apparent effect was observed in the samples due to the height of the samples.

### 3.4. Colour Changes

Color changes in the doughs and the baked samples on the crust and crumb were analyzed. Table 3 shows changes in color parameters. Regarding the doughs, the control dough showed central coordinates and high luminosity values, which were attributed to a white achromatic sample, as shown in Figure 3. Rosehip increased a* and b* coordinates and decreased luminosity, providing an orange hue. The samples with encapsulated rosehip showed intermediate values between the rosehip and control doughs due to the maltodextrin bleaching the sample, as shown in Figure 3. Both samples show appreciable differences concerning the control since they were higher than three units (i.e., the human perceptible limit) [51].

After baking, it has been observed that rosehip reduces the brightness (although not significantly (*p* > 0.05) when the sample has 1 cm of height). As rosehip is rich in carotenoids [38], samples with rosehip had higher a* coordinate values. Those containing maltodextrin also had intermediate values between the control and the rosehip samples due to the white-brown hue of maltodextrin. In all cases, the addition of compounds provided visually appreciable differences concerning the control sample.

Regarding the values obtained from the breadcrumbs, the control samples were the ones with the brightest color. These samples had a light yellowish hue. In those where rosehip was added, the values showed that the color was slightly less luminous and orange in hue. The human eye could appreciate the color differences (ΔE) when rosehip was incorporated.

### 3.5. Texture

Texture is one of the most relevant aspects of developing baking products. TPA test was performed on the crumb and a puncture test on the crust to evaluate the texture of the baked samples.

Table 4 shows the different parameters of the TPA test, and the maximum force obtained in the puncture test for the different samples. The crumb that requires the most significant force for deformation was the one corresponding to the 3 cm height control sample, although it was not significantly (*p* > 0.05) different from the 2 cm height control sample. The adhesiveness of the samples was negligible. The cohesiveness of the samples was high, in some cases reaching 87% deformation to cause crumb breakage. The most cohesive samples were that incorporated encapsulated rosehip and rosehip. The addition of rosehip generally makes the product more resistant to breakage, which could be due to the fiber content of the rosehip.

Regarding the springiness of the doughs, it was observed that most of the samples recovered almost entirely in their initial state, reflecting the elastic behavior of the samples. The sample with a lower springiness value was the 1 cm height control. In terms of chewiness, the 3 cm height sample with rosehip and the control were the ones that would have to be chewed the most to be able to swallow. They did not differ significantly (*p* > 0.05) from the 3 cm height sample with encapsulated rosehip and the 2 cm height control. Resilience is the parameter that refers to how the sample recovers from deformation in terms of time and strength. It was observed that the samples that take the least time to recover their initial state incorporate rosehip, both encapsulated and unencapsulated.

The puncture test showed the force required to penetrate the crust of the samples. The sample requiring the most force was the 1 cm height control, followed by 2 cm height control and 3 cm height control (encapsulated).

From the texture parameters analysis, it could be said that the effect of height was more pronounced than the effect of the different compositions of the samples [16].

### 3.6. Centesimal Composition

In new product development, it was essential to determine the product’s composition, either to understand the behavior/expression of the product and to evaluate its nutritional value. The rosehip incorporation in gluten-free bread improves the product, approaching the nutritional needs of the celiac population. The nutritional data (carbohydrates, fiber, fat, protein, and ashes) for each sample are presented in Table 5.

Carbohydrates characterized samples as the primary macronutrient. The samples with the highest available carbohydrate content were the control samples, followed by the samples with encapsulated rosehip. Rosehip addition reduced available carbohydrates but increased fiber content. The second major component of the samples was water (Table 2). As mentioned above, the water content increased with the incorporation of rosehips due to retention by the fiber. The samples did not show significant (*p* > 0.05) differences by composition in terms of fat content, but the sample height was affected. The higher the sample, the lower the fat content. No effect was observed in protein content in all formulations.

### 3.7. Bioactive Compounds

Bioactive compounds have numerous beneficial functions for the organism, as is the case of carotenoids or phenols which have antioxidant capacities [18]. Rosehips are rich in carotenoids, phenolic compounds, and vitamin C [52]. The powdered rosehip obtained by freeze-drying has high contents of these compounds, and this powder has been incorporated as ingredients to obtain other products such as snacks [20].

Table 6 shows the bioactive content (total phenols and carotenoids) and antioxidant capacity for doughs and printed baked samples. It could be seen in the doughs that incorporation of rosehip increases the number of total phenols by five times and the number of total carotenoids by about fifty times. The antioxidant capacity has a lower increase due to the ascorbic acid included in the ingredients of the baking mix. These increases are reduced when incorporating encapsulated rosehip due to the lower proportion of rosehip in the mix.

After baking, it was observed that the samples with the highest total phenol content were those containing only rosehip. Samples with encapsulated rosehip showed values close to the samples with only rosehip but lower degradation percentages (67% for sample 1RB and 37% for sample 1RMB). A protective effect was also observed related to the sample’s height, with the highest degradations occurring at heights of 1 cm.

The same behavior was observed for total carotenoids and total phenols. The samples with the highest values incorporated rosehip and had the highest losses. Samples with encapsulated rosehip had lower degradation percentages, 49.6% in 3RB compared to 33.5% in 3RMB. These results are similar to those obtained in previous works [21] that incorporated rosehip in extruded products. In this work, samples with the highest carotenoid values incorporated encapsulated rosehip, obtaining lower degradation percentages.

The same behavior was observed in the antioxidant capacity. The samples with the highest values were those incorporating only rosehip, followed by those with encapsulated rosehip. All samples showed a similar degradation percentage regarding the loss of these components due to baking.

Correlation statistical analyses were performed to explain the relationships in AC with CT and TP. CT and TP showed a significant (*p* < 0.05) positive Pearson’s correlation coefficient with AC. TC played the highest role in the AC of doughs and bread (0.9250), followed by total phenols (0.8088). This trend was similar to the obtained in other works with corn extrudates with rosehip added [21].

## 4. Conclusions

Food 3D printing is strongly affected by the input food rheology. In this study, the impact of rheology of the masses studied on the stability of the 3D figure can be observed. The three doughs used to obtain 3D printed gluten-free breads presented good definition of the printing lines. However, their rheological properties do not allow printing 3D figures with heights of 3 cm without noticeable deformations. In this sense, knowing the characteristic rheological parameters allows us to establish relationships with food printability and the stability of printed figures. The enrichment of gluten-free bread with rosehips did not show effects on the texture, but it did show changes in the hue of the crumb towards orange colors. Moreover, the incorporation of rosehip, encapsulated and unencapsulated, increased the product’s nutritional value. The rosehip provided fiber and bioactive compounds such as phenols and carotenoids with antioxidant properties essential for human health.

This study is an incipient proposal for the use of 3D printing in bread dough as a food personalization technique. This area still requires many complementary studies to refine the technique and its multiple uses.

## Figures and Tables

**Table 1:** Rheological parameters of the doughs (C, control; R, rosehip; RM, encapsulated rosehip).

Dough	G′ (Pa)	G″ (Pa)	tan δ	η* (Pa s)
C	1408 ± 64 ^a^	324 ± 14 ^a^	0.2300 ± 0.0003 ^a^	230 ± 10 ^a^
R	1639 ± 88 ^b^	410 ± 19 ^b^	0.2499 ± 0.0018 ^c^	269 ± 14 ^b^
RM	1409 ± 92 ^a^	339 ± 21 ^a^	0.2408 ± 0.0011 ^b^	231 ± 15 ^a^

The letters (a–c) in columns indicate homogeneous groups according to ANOVA (*p* < 0.05).

**Table 2:** Physico-chemical parameters of doughs and breads (C, control; R, rosehip; RM, encapsulated rosehip; 1, 2, 3 cm height; D, dough; B, bread).

Sample	a_w_	Moisture (g/100 g Sample)
CD	-	58.9 ± 0.2 ^a^
RD	-	58.62 ± 0.13 ^a^
RMD	-	57.1 ± 0.7 ^b^
1CB	0.8850 ± 0.0017 ^a^	26.6 ± 0.7 ^c^
1RB	0.906 ± 0.002 ^b^	35.434 ± 0.014 ^e^
1RMB	0.924 ± 0.003 ^e^	39.6 ± 0.4 ^g^
2CB	0.918 ± 0.0012 ^d,e^	31.6 ± 0.4 ^d^
2RB	0.919 ± 0.007 ^d,e^	40.4 ± 0.2 ^g^
2RMB	0.908 ± 0.012 ^b,c^	38.0 ± 0.8 ^f^
3CB	0.913 ± 0.009 ^b,c,d^	36.1 ± 0.9 ^e^
3RB	0.918 ± 0.003 ^c,d,e^	40.5 ± 0.8 ^g^
3RMB	0.926 ± 0.007 ^e^	39.78 ± 0.04 ^g^

The letters (a–g) in columns indicate homogeneous groups according to ANOVA (*p* < 0.05). (C, control; R, rosehip; RM, encapsulated rosehip; D, dough; B, bread; 1, 2, 3 cm height).

**Table 3:** Color parameters of the samples for a D65 illuminant and 10° observer (D, dough; Ct, crust; Cb, crumb B control; E, rosehip; EM, rosehip maltodextrin; 1, 2, 3 cm height).

Sample	L*	a*	b*	h	C	ΔE
CD	81.221 ± 0.019 ^c^	−1.7 ± 0.2 ^a^	9 ± 0.2 ^a^	100.5 ± 0.3 ^c^	9 ± 0 ^a^	-
RD	64.1 ± 0.3 ^a^	14.1 ± 0.4 ^c^	29.2 ± 0.4 ^c^	64.2 ± 0.3 ^a^	32.4 ± 0.6 ^c^	30.8 ± 0.6 ^b^
RMD	66.6 ± 0.2 ^b^	10.6 ± 0.3 ^b^	24.7 ± 0.3 ^b^	66.7 ± 0.5 ^b^	26.9 ± 0.3 ^b^	24.7 ± 0.3 ^a^
1C Ct	59 ± 8 ^c,d^	7.1 ± 1.8 ^b^	25 ± 6 ^c,d^	73 ± 7 ^d^	26 ± 5 ^b,c^	-
1R Ct	60 ± 7 ^c,d^	7.7 ± 0.8 ^b,c^	24 ± 2 ^c,d^	72 ± 3 ^d^	25.5 ± 1.9 ^b,c^	11 ± 6 ^a^
1RM Ct	65 ± 5 ^d^	5.3 ± 1.4 ^a^	20 ± 3 ^a,b,c^	75 ± 4 ^d^	21 ± 3 ^a,b^	11 ± 8 ^a^
2C Ct	64 ± 3 ^d^	7.4 ± 0.8 ^b,c^	28 ± 2 ^d^	75.3 ± 1.6 ^d^	29 ± 2 ^c^	-
2B Ct	47 ± 8 ^a,b^	10.4 ± 1.5 ^e^	17 ± 8 ^a,b^	55 ± 12 ^a,b^	20 ± 7 ^a,b^	21 ± 10 ^b^
2RM Ct	54 ± 7 ^b,c^	8.6 ± 1.5 ^c,d^	22 ± 5 ^b,c,d^	68 ± 4 ^c,d^	24 ± 5 ^a,b,c^	13 ± 8 ^a^
3C Ct	62 ± 5 ^d^	6.9 ± 1.2 ^b^	27 ± 4 ^d^	75 ± 3 ^d^	28 ± 4 ^c^	-
3R Ct	46 ± 9 ^a^	10 ± 2 ^d,e^	15 ± 10 ^a^	48 ± 9 ^a^	19 ± 9 ^a^	21 ± 10 ^b^
3RM Ct	50 ± 10 ^a,b^	8.8 ± 1.6 ^c,d^	19 ± 9 ^a,b,c^	59 ± 19 ^b,c^	22 ± 8 ^a,b^	16 ± 14 ^a,b^
1C Cb	50 ± 4 ^b^	1.4 ± 0.9 ^a^	7.5 ± 0.9 ^a^	79 ± 7 ^d^	7.7 ± 0.9 ^a^	-
1R Cb	47.0 ± 1.3 ^a^	12.4 ± 1.2 ^e^	23.1 ± 1.6 ^d^	61.8 ± 1.3 ^c^	26.3 ± 1.8 ^e^	20 ± 2 ^e^
!RM Cb	45 ± 5 ^a^	8.1 ± 0.7 ^b^	14 ± 3 ^b^	59 ± 5 ^b,c^	16 ± 2 ^b^	12 ± 3 ^a^
2C Cb	56 ± 3 ^c^	0.6 ± 0.9 ^a^	9 ± 2 ^a^	86 ± 4 ^e^	9 ± 2 ^a^	-
2R Cb	44 ± 3 ^a^	11.7 ± 1.2 ^e^	17 ± 4 ^c^	54 ± 4 ^a^	20 ± 3 ^c^	18 ± 3 ^d,e^
2RM Cb	47 ± 3 ^a^	9 ± 1.2 ^b,c^	15 ± 3 ^b,c^	59 ± 2 ^b,c^	18 ± 4 ^b,c^	15 ± 3 ^b,c^
3C Cb	55.0 ± 0.8 ^c^	1 ± 2 ^a^	9 ± 4 ^a^	85 ± 6 ^e^	9 ± 4 ^a^	-
3R Cb	44.1 ± 1.5 ^a^	10.3 ± 1.5 ^d^	16 ± 2 ^b,c^	57 ± 3 ^a,b^	19 ± 3 ^c^	17 ± 3 ^c,d^
3RM Cb	47 ± 2 ^a^	9.6 ± 0.8 ^c,d^	16 ± 2 ^b,c^	59 ± 2 ^b,c^	18 ± 2 ^b,c^	14 ± 3 ^a,b^

The letters (a–e) in columns indicate the homogeneous groups between same sample parts according to ANOVA (*p* < 0.05). (D, dough; Ct, crust bread; Cb, crumb bread; C, control; R, rosehip; RM, encapsulated rosehip; 1, 2, 3 cm height).

**Table 4:** TPA test parameters and crust puncture.

Sample	H (N)	A (N·s)	C	S	Ch (N)	R	P (N)
1CB	0.64 ± 0.12 ^a,b^	−0.009 ± 0.008 ^a^	0.59 ± 0.06 ^a^	0.65 ± 0.12 ^a^	0.25 ± 0.09 ^a^	0.32 ± 0.04 ^a^	3.9 ± 0.6 ^f^
1RB	0.42.6 ± 0.012 ^a^	−0.06 ± 0.05 ^a^	0.73 ± 0.16 ^b,c^	0.92 ± 0.05 ^b,c^	0.29 ± 0.08 ^a^	0.38 ± 0.06 ^b^	2.9 ± 1.3 ^e^
1RMB	1.19 ± 0.21 ^a,b,c^	−0.012 ± 0.017 ^a^	0.84 ± 0.03 ^d^	0.924 ± 0.014 ^c^	0.93 ± 0.17 ^b,c^	0.525 ± 0.009 ^c^	2.4 ± 0.5 ^d,e^
2CB	2.18 ± 0.26 ^d,e^	−0.09 ± 0.19 ^a^	0.69 ± 0.06 ^a,b^	0.88 ± 0.03 ^b,c^	1.4 ± 0.2 ^c,d^	0.36 ± 0.04 ^a,b^	2.4 ± 0.6 ^d,e^
2RB	1.33 ± 0.31 ^b,c^	−0.04 ± 0.03 ^a^	0.82 ± 0.04 ^c,d^	0.93 ± 0.02 ^c^	1.03 ± 0.23 ^b,c^	0.49 ± 0.04 ^c^	2.2 ± 0.8 ^c,d^
2RMB	0.93 ± 0.11 ^a,b^	0 ± 0 ^a^	0.818 ± 0.019 ^c,d^	0.93 ± 0.04 ^c^	0.71 ± 0.11 ^b^	0.5 ± 0.02 ^c^	1.8 ± 0.7 ^b,c^
3CB	2.71 ± 0.91 ^e^	−0.02 ± 0.02 ^a^	0.71 ± 0.04 ^b^	0.85 ± 0.04 ^b^	1.6 ± 0.4 ^d^	0.38 ± 0.04 ^b^	1.6 ± 0.2 ^b^
3RB	1.85 ± 0.65 ^c,d^	−0.006 ± 0.008 ^a^	0.84 ± 0.03 ^c,d^	0.959 ± 0.003 ^c^	1.5 ± 0.6 ^d^	0.508 ± 0.013 ^c^	1.2 ± 0.4 ^a,b^
3RMB	1.38 ± 0.14 ^b,c,d^	−0.02 ± 0.03 ^a^	0.88 ± 0.03 ^d^	0.9555 ± 0.0009 ^c^	1.15 ± 0.08 ^b,c,d^	0.5427 ± 0.0002 ^c^	0.74 ± 0.18 ^a^

The letters (a–f) in columns indicate the homogeneous groups according to ANOVA (*p* < 0.05). (H, hardness; A, adhesiveness; C, cohesiveness; S, springiness; Ch, chewiness; R, resilience; Fp, maximum puncture force; CB, control bread; RB, rosehip bread; RMB, encapsulated rosehip bread; 1, 2, 3 cm sample height).

**Table 5:** Centesimal composition of the different samples expressed in g/100 g sample.

Sample	Available Carbohydrates	Fiber	Fat	Protein	Ashes
1CB	61 ± 2 ^d^	4.9 ± 1.2 ^b^	3.66 ± 0.03 ^f^	0.74 ± 0.06 ^c^	2.94 ± 0.12 ^b,c^
1RB	42.98 ± 0.19 ^a^	14.5 ± 0.3 ^e^	3.5 ± 0.2 ^e,f^	0.72 ± 0.07 ^c^	2.89 ± 0.12 ^b^
1RMB	48.6 ± 1.8 ^b^	5.4 ± 1.2 ^b^	3.31 ± 0.16 ^e^	0.58 ± 0.05 ^a,b^	2.51 ± 0.07 ^a^
2CB	54.9 ± 1.3 ^c^	7.9 ± 0.6 ^c^	2.54 ± 0.12 ^c,d^	0.57 ± 0.04 ^a,b^	2.48 ± 0.12 ^a^
2RB	41.1 ± 0.6 ^a^	12.88 ± 0.12 ^e^	2.42 ± 0.05 ^c^	0.61 ± 0.06 ^b^	2.63 ± 0.18 ^a^
2RMB	53.75 ± 0.06 ^c^	2.3 ± 0.6 ^a^	2.74 ± 0.03 ^d^	0.551 ± 0.007 ^a,b^	2.67 ± 0.12 ^a^
3CB	54 ± 2 ^c^	3.8 ± 1.2 ^a,b^	1.94 ± 0.15 ^a,b^	0.50 ± 0.04 ^a^	3.156 ± 0.009 ^c^
3RB	43.0 ± 1.2 ^a^	10.4 ± 0.2 ^d^	2.12 ± 0.04 ^b^	0.590 ± 0.004 ^a,b^	3.499 ± 0.006 ^d^
3RMB	49.9 ± 0.6 ^b^	4.3 ± 0.6 ^b^	1.71 ± 0.05 ^a^	0.55 ± 0.04 ^a,b^	3.788 ± 0.008 ^e^

The letters (a–f) in columns indicate the homogeneous groups according to ANOVA (*p* < 0.05). (CB, control bread; RB, rosehip bread; RMB, encapsulated rosehip bread; 1, 2, 3 cm sample height).

**Table 6:** Result of the bioactive content expressed in mg/100 g dry sample for; TP, total phenols; AC, antioxidant capacity; TC, total carotenoids).

Sample	TP	TC	AC (Teq)
CD	27.0 ± 0.9 ^a^	0.32 ± 0.02 ^a^	75 ± 5 ^b,c^
RD	145 ± 4 ^f^	15.33 ± 0.09 ^h^	136 ± 6 ^f^
RMD	77.39 ± 0.04 ^d,e^	7.23 ± 0.08 ^e^	92.0 ± 1.8 ^d^
1CB	26.7 ± 1.2 ^a^	0.32 ± 0.04 ^a^	59 ± 2 ^a^
1RB	46.5 ± 0.7 ^b^	7.998 ± 0.018 ^g^	106.1 ± 1.5 ^e^
1RMB	48 ± 3 ^b^	5.22 ± 0.02 ^d^	79 ± 3 ^c^
2CB	24.9 ± 1.2 ^a^	0.32 ± 0.02 ^a^	57.9 ± 0.3 ^a^
2RB	77 ± 2 ^c,d,e^	7.776 ± 0.013 ^f^	112 ± 3 ^e^
2RMB	71.9 ± 0.9 ^c^	4.55 ± 0.03 ^b^	74 ± 4 ^b,c^
3CB	27.0 ± 0.4 ^a^	0.383 ± 0.012 ^a^	66.2 ± 1.8 ^a,b^
3RB	81 ± 3 ^e^	7.72 ± 0.02 ^f^	113.5 ± 1.2 ^e^
3RMB	73 ± 4 ^c,d^	4.81 ± 0.08 ^c^	71 ± 9 ^b,c^

The letters (a–h) in columns indicate the homogeneous groups according to ANOVA (*p* < 0.05). (C: control; R, rosehip; RM, encapsulated rosehip; D, dough; B, bread; 1, 2, 3 cm height).

## Data Availability

Data is contained within the article.
